# From Emotional Intelligence to Self-Injuries: A Path Analysis in Adolescents With Conduct Disorder

**DOI:** 10.3389/fpsyt.2020.556278

**Published:** 2021-01-08

**Authors:** Joanna Halicka-Masłowska, Monika Szewczuk-Bogusławska, Joanna Rymaszewska, Agnieszka Adamska, Błażej Misiak

**Affiliations:** Department of Psychiatry, Wroclaw Medical University, Wroclaw, Poland

**Keywords:** emotional intelligence, self-harm, self-injuries, conduct disorder, adolescent

## Abstract

**Objective:** Self-harm acts are highly prevalent among adolescents with conduct disorder. It has been shown that low level of emotional intelligence (EI) might be related to a higher risk of self-injuries. However, the exact mechanisms underlying this association are still unclear. The purpose of this study was to explore whether psychopathological symptoms and selected psychological processes mediate the association between EI and self-harm risk in adolescents with conduct disorders.

**Method:** Out of 162 adolescents with conduct disorder approached for participation, 136 individuals (aged 14.8 ± 1.2 years, 56.6% females) were enrolled and completed the questionnaires evaluating the level of EI, depression, anxiety, impulsiveness, empathy, venturesomeness, self-esteem, and disgust.

**Results:** Individuals with a lifetime history of self-injuries had significantly higher levels of depression, anxiety and impulsivity as well as significantly lower levels of EI and self-esteem. Higher levels of EI were associated with significantly higher levels of self-esteem, venturesomeness and empathy as well as significantly lower levels of depression, anxiety and impulsivity. Further analysis revealed that trait and state anxiety as well as self-esteem were complete mediators of the association between EI and self-harm risk.

**Conclusions:** Our findings indicate that anxiety and self-esteem might mediate the association between EI and a risk of self-injuries in adolescents with conduct disorder. However, a cross-sectional design of this study limits conclusions on the direction of causality. Longitudinal studies are needed to test validity of our model.

## Introduction

Non-suicidal self-injuries (NSSI) are increasingly being recognized as a highly prevalent aspect of psychopathology in young people. Recent epidemiological studies have shown that self-harm occurs in 17–18% of adolescents in the general population (1) and 40–80% of psychiatric patients (2). It has been estimated that even 92% of people consulted at the general hospital due to self-injuries might have one or more mental disorders (3, 4). Self-injuries are listed among the diagnostic criteria for borderline personality disorder [DSM-5; (5)]; however, they can appear in patients with other mental disorders. The Diagnostic and Statistical Manual Version 5 (DSM-5) (5) has pointed out “non-suicidal self-injury disorder” (NSSID) as a problem to further study that extends current diagnostic boundaries (1). According to the International Society for the Study of Self-Injury, NSSI can be defined as the deliberate, self-inflicted damage to body tissue without suicidal intent and for purposes not sanctioned by society or culture (6). It has been reported that self-injuries might be associated with a number of negative outcomes that include repetitive self- injuries (7) and suicide (8).

It is now widely accepted that self-harm may occur in the context of various mental disorders and psychopathological symptoms. To date, several mental disorders that might develop in adolescence have been associated with self-harm risk, including attention deficit hyperactivity disorder, anxiety and depressive disorders, and conduct disorder (9). It has been noted that depression is a risk factor for self-harm, with affective disorders, such as bipolar disorder and depression being the most common primary diagnoses of patients who engage in self-harm acts (72%) and commit suicide (45%) (3, 10). Based on a meta-analysis, Fox et al. (11) found that the possibility of externalizing disorder symptoms is higher than the one of internalizing disorder symptoms among individuals who engage in non-suicidal self-injuries. The study by Nock et al. (12) estimated the prevalence of any externalizing disorder at 62.9%, and the presence of any internalizing disorder at 51.7% in adolescents who engage in self-harm the prevalence. In turn, prevalence rates of self-harm acts in adolescents with conduct disorder have been estimated at 15.5–62.5% (13). For instance, our group has recently reported that almost 53% of adolescent girls with conduct disorder have a history of self-injuries (14).

It has been shown that emotional intelligence (EI) can be associated with a risk of self-harm. Indeed, EI provides effective ways of balancing negative affect in adolescence and protecting from the aftermaths of self-harm. According to Goleman (15), it is a set of social skills that refer to the capacity to understand own emotions, manage and control them as well as the ability to empathize. EI may be perceived as a tool to encompass a personality dimension and also as the means to comprehend, process, and use affect-laden information gained by monitoring other's and one's own emotions. EI relies upon the ability to take suitable action to overcome the problem (16).

It has been reported that lower EI is associated with higher risk of internalizing disorders, including depression and anxiety, as well as substance use and less efficient coping (17). Petrides and Furnham (18) reported that in people with a higher level of EI, it serves as a protective factor for suicidal attempts and ideation (17). However, emerging evidence indicates that EI is not directly associated with suicide risk. The recent study by Quintana-Orts et al. (19) showed that depressive symptoms mediate the association between low level of EI and suicide risk among people who were bullied. This mediation appeared to be stronger among girls. At least theoretically, other processes might also mediate the association between EI and self-harm risk.

Individuals engaging in self-harm experience a variety of negative emotions. The most common categories of unpleasant emotional states declared by these individuals include feelings of guilt, anger, frustration, fear, sadness, shame, tension, anxiety and contempt (20). Apart from these emotions, there is evidence that disgust often occurs in this group of individuals, and in contrast to most other emotions, it does not tend to decrease after self-harming. It can be recognized as one of trait-dependent aspects of those who are prone to engage in self-harm acts (21). Another important aspect connected to self-harm is “impulsivity.” It refers to actions that are risky, unduly hasty, and damaging (22). Higher levels of impulsivity have been reported in subjects with a history of self-harm (23). Moreover, higher levels of impulsivity and aggression have been associated with lower levels of EI (24). Finally, there is evidence that lower self-esteem might be related to higher risk of self-harm. In this regard, self-dislike in adolescents can be perceived as the way of punishing oneself and developing self-injurious behaviors (25). On the other hand, a significant positive relationship between the levels of EI and self-esteem has been demonstrated (26).

A majority of previous studies have investigated single correlates of psychological constructs associated with EI and self-harm. In light of findings mentioned above, we aimed to investigate as to whether psychopathology and selected psychological processes mediate the association between EI and self-harm risk in adolescents with conduct disorder. More specifically, we tested the hypothesis that depressive and anxiety symptoms, aggression, impulsivity, self-esteem as well as disgust mediate this association in adolescents with conduct disorder.

We decided to focus on adolescent patients due to the highest prevalence of NSSI among people at this age. A broad spectrum of negative emotions leading to aggressive behavior is typical for conduct disorder. One of the key functions of NSSI is to relieve negative feelings. Thus, we decided to assess this specific group of patients because of co-occurrence of NSSI and emotional dysregulation which play important roles as triggers of NSSI.

## Method

### Participants

Participants were enrolled among the students of the Youth Sociotherapy Centre (YSTC) No. 2 in Wroclaw, Poland. YSTCs in Poland have been designed by the Ministry of National Education to provide comprehensive pedagogical, educational and psychological support for children and adolescents with different problems or disorders (developmental, learning or social) who are at risk of social maladjustment. Adolescents, being admitted to the YSTC No 2 in Wrocław (Poland), mainly present with conduct disorder (mild or moderate severity of symptoms). Residents of YSTCs receive accommodation and attend school at these facilities. Students are recruited to YSTCs based on the opinion stating special education needs issued by professionals from the psychological and pedagogical counseling centers. According to the DSM-V criterion F of non-suicidal self-injury disorder (NSSID), participants were excluded if they had presented with intellectual disability, delirium, intoxication or withdrawal symptoms, psychotic disorder or autism spectrum disorders. Out of 162 individuals approached for participation (all individuals residing in the YSTC at the time of the study), 144 adolescents were enrolled (3 individuals and/or their legal guardians refused to participate and 15 individuals were transferred to another institution). Due to a lack of necessary data to perform analyses, eight participants were excluded. The final sample included 136 adolescents (77 females and 59 males).

### Procedures

The data were collected from September 2016 to August 2019 by a psychologist and a psychiatrist. Taking care of the comfort of the subjects, the study was divided into three parts, each lasting about an hour. During the first part, data on self-inflicted injuries were collected. A semi-structured questionnaire was administered to confirm a history of self-harm. This questionnaire recorded the information regarding the frequency of self-injuries and suicidal behaviors (suicidal thoughts and attempts) that had occurred at different time periods (lifetime as well as the preceding year, month, and week).

During the second part, all participants underwent psychiatric examination using the MINI-Kid interview. The MINI-Kid is a structured diagnostic tool, developed together by European and American psychiatrists and clinicians, for the DSM-IV and the ICD-10 criteria (27). This measure was used to establish a diagnosis of conduct disorder and comorbid mental disorders. Apart from the MINI-Kid, a diagnosis of CD was confirmed based on participants' psychiatric examination, medical records and psychological opinion. Furthermore, a diagnosis of potential comorbid mental disorders listed as exclusionary diagnoses of NSSID in the DSM-5 (criterion F), except for intellectual disability, was carried out. All students were assessed regarding intellectual functions before admission to YSTC by psychologists from the psychological and pedagogical counseling centers. After psychiatric examination, participants were divided into two groups – adolescents with a positive lifetime history of self-injuries and those who had never engaged in self-harm acts. We decided to focus our analyses on this categorization due to controversies around operationalization of the severity of self-injuries. For instance, although the NSSID has been developed in the DSM-5 as a new diagnostic category for further studies, there are studies showing insufficient validity of the NSSID frequency criterion (1, 14).

During the third part, emotional intelligence and concomitant psychopathology were assessed. Questionnaire data regarding self-esteem, impulsivity, depressive symptoms, anxiety and aggression levels and disgust sensitivity were collected using standardized self-reports. Self-reports were administered in the following order: (1) The Popular Emotional Intelligence Questionnaire (PEIQ); (2) The Buss-Perry Aggression Questionnaire (BPAQ); (3) The Children's Depression Inventory 2 (CDI2); (4) The State-Trait Anxiety Inventory (STAI); (5) The Rosenberg Self-Esteem Scale (SES); (6) The Eysenck's Impulsivity Inventory (IVE) and (7) The Questionnaire for the Assessment of Disgust Sensitivity (QADS).

The study was approved by the Bioethics Committee of Wroclaw Medical University, Poland. All participants and their statutory representatives gave written consent to all procedures carried out as the part of this study.

### Self-Report Measures

#### The Popular Emotional Intelligence Questionnaire (PEIQ)

It measures EI and consists of 94 items of self-descriptive nature, using a five-point Likert scale. The PEIQ consists of the following subscales: acceptance (expressing and using own emotions), empathy (understanding and recognizing emotions of other people), control (control over one's emotions), and understanding (understanding and awareness of own emotions) (28). The Cronbach's alpha for the PEIQ was estimated at 0.89 in our sample.

#### The Buss-Perry Aggression Questionnaire (BPAQ)

The BPAQ is a self-report measure of aggression in adolescents and adults. The BPAQ has 29 items, subdivided into four factors: physical aggression, verbal aggression, anger, and hostility (29). The Cronbach's alpha for the BPAQ total score in our sample was 0.80, for physical aggression 0.77, for verbal aggression 0.73, for anger 0.62 and for hostility 0.77.

#### The Children's Depression Inventory 2 (CDI2)

This measure includes 28 items. It is a measure which allows for a comprehensive assessment of depressive symptoms in children and adolescents. The questionnaire also includes scales measuring emotional problems and problems related to everyday functioning. In addition, the self-rating version includes subscales measuring negative mood/somatic symptoms, low self-esteem, lack of behavior efficacy, interpersonal problems, emotional problems and problems in functioning (30). The Cronbach's alpha for the CDI2 was 0.94 in our sample.

#### The State-Trait Anxiety Inventory (STAI)

This measure consists of two subscales measuring anxiety as a relatively stable personality component (state anxiety subscale) and the level of transient anxiety attributable to specific situations (trait anxiety subscale). Each subscale consists of 20 items which the subject answers by selecting one of four pre-categorized answers (31). The Cronbach's alpha for our sample was 0.94 for state anxiety and 0.99 for trait anxiety.

#### The Rosenberg Self-Esteem Scale (SES)

This tool consists of 10 diagnostic questions. Each question is based on a four-point Likert scale illustrating the level of agreement with the statements. The SES is a one-dimension tool which measures the level of overall self-esteem—approximately consistent disposition understood as conscious attitude—positive or negative toward the self (32). The Cronbach's alpha for the SES total score in our sample was 0.89.

#### The Eysenck's Impulsivity Inventory (IVE)

This measure consists of 63 diagnostic questions, using a two-point scale. The IVE consists of the following subscales: impulsivity, venturesomeness, and empathy (33). The Cronbach's alpha for each subscale was as follows: 0.75 (for impulsivity), 0.66 (for venturesomeness), and 0.65 (for empathy).

#### The Questionnaire for the Assessment of Disgust Sensitivity (QADS)

This measure consists of 37 statements, in which the severity of disgust is assessed on a Likert five-point scale. Disgust sensitivity refers to individual personality traits and describes a predisposition to react to specific situations and materials with disgust. There are three subscales in the questionnaire: Core Disgust, Animal-Reminder, and Contamination-Interpersonal (34). Animal – Reminder disgust sensitivity addresses these aspects of human functioning which are shared with animals i.e., death, sex, a lack of hygiene, and damage to the body surface. The Cronbach's alpha for the QADS total score in our sample was 0.94.

### Statistical analysis

**Figure 1:**
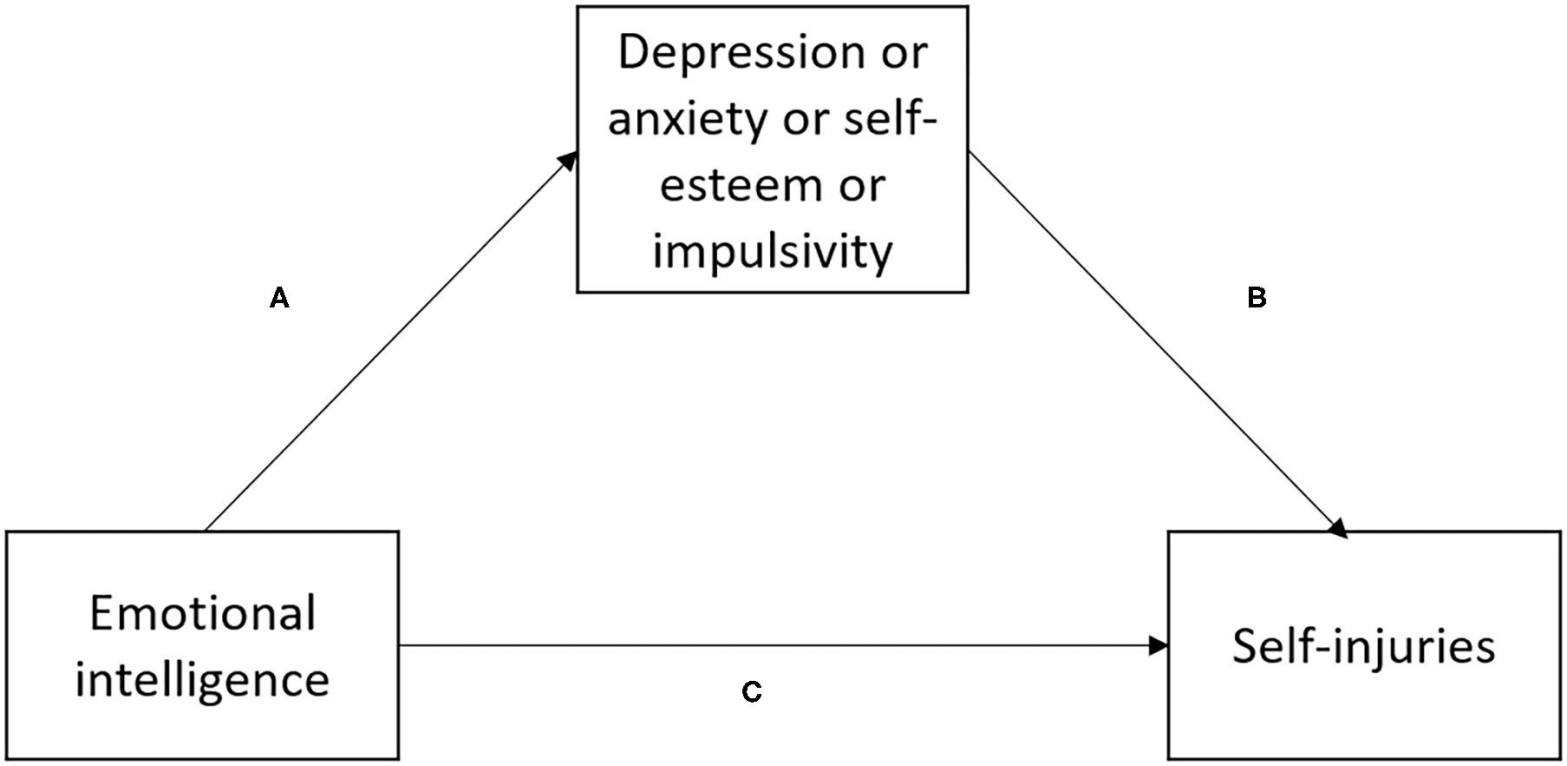
A simple mediation model tested in this study. **(A)** effect of emotional intelligence on mediator, **(B)** effect of mediator on self-injuries, **(C)** direct effect of emotional intelligence on self-injuries.

The chi^2^ test was applied to evaluate sex differences as well as differences in the rates of comorbid mood and anxiety disorders between participants with lifetime history of self-ham and those who did not engage in self-harm acts. Due to non-normal distribution, the Spearman rank correlation coefficients and the Mann-Whitney U test were used to analyze continuous variables. Results of bivariate tests were considered statistically significant if their *p*-value was <0.05. Simple mediation was analyzed using the PROCESS Macro Model 4 (35). Separate models for specific mediators were analyzed to avoid potential multicollinearity (Figure 1). The PEIQ score was inputted as an independent variable while a history of self-injuries was an outcome variable. One of main assumptions underlying mediation analysis is that the mediator must be associated with the independent variable and the outcome variable. Therefore, potential mediators were selected from the measures that were significantly associated with the PEIQ score and lifetime history of self-injuries. Age and sex were added as co-variates. The bootstrap calculation with 5,000 samples was applied to check direct and indirect effects. Mediation was considered significant if the 95% CI of indirect effect did not include zero. All analyses were conducted using the Statistical Package for Social Sciences, version 20 (SPSS Inc., Chicago, Illinois, USA).

## Results

The comparison of adolescents with a positive history of self-harm and those who had never engaged in self-injuries was provided in Table 1. Females were overrepresented in the subgroup of adolescents who reported engaging in self-injuries. Individuals with a positive history of self-harming presented with significantly lower levels of EI (PEIQ – total score and scores of acceptance, control and understanding) and self-esteem as well as significantly higher levels of depression, state and trait anxiety and impulsivity.

**Table 1:** General characteristics of the sample.

	**Self-harm (+) *n* = 78**	**Self-harm (–), *n* = 58**	**Statistics**
Age, years	14.6 ± 1.1	15.1 ± 1.3	*U* = 1,814.5, *r* = −0.16, *p* = 0.055
Sex, F/M (%)	57/21 (73.1/26.9)	20/38 (34.5/65.5)	**χ^2^ = 20.2**, ***p*** **< 0.001**
Age of self-harm onset, years	10.7 ± 4.4	–	–
Lifetime number of self-harm acts	179.3 ± 362.5	–	–
The number of self-injuries in the preceding year	35.8 ± 77.6	–	–
Comorbid mood and/or anxiety disorder, *n* (%)	28 (35.9%)	12 (20.7)	χ^2^ = 3.71, *p* = 0.054
CDI2 – depression	20.4 ± 13.4	12.9 ± 11.0	***U*** **= 601.5**, ***r*** **= 0.29**, ***p*** **= 0.024**
STAI – trait anxiety	47.0 ± 13.2	36.8 ± 9.5	***U*** **= 3,120.5**, ***r*** **= 0.46**, ***p*** **< 0.001**
STAI – state anxiety	50.3 ± 12.7	37.7 ± 8.9	***U*** **= 3,328.5**, ***r*** **= 0.55**, ***p*** **< 0.001**
PEIQ – EI (total score)	297.0 ± 29.2	309.8 ± 33.2	***U*** **= 1,585.0**, ***r*** **= −0.21**, ***p*** **= 0.022**
PEIQ – acceptance	46.0 ± 9.0	50.1 ± 10.7	***U*** **= 1,563.0**, ***r*** **= −0.21**, ***p*** **= 0.017**
PEIQ – empathy	65.1 ± 12.1	61.9 ± 10.4	*U* = 2,400.0, *r* = 0.14, *p* = 0.123
PEIQ – control	30.0 ± 6.8	33.8 ± 5.6	***U*** **= 1,301.5**, ***r*** **= −0.32**, ***p*** **< 0.001**
PEIQ – understanding	27.3 ± 6.9	29.9 ± 4.8	***U*** **= 1,535.0**, ***r*** **= −0.22**, ***p*** **= 0.011**
SES – self-esteem	23.8 ± 6.4	28.6 ± 5.4	***U*** **= 1,188.0**, ***r*** **= −0.35**, ***p*** **< 0.001**
QADS - disgust (total score)	123.1 ± 31.5	118.4 ± 32.6	*U* = 2,209.5, *r* = 0.06, *p* = 0.457
QADS – core disgust	54.5 ± 15.2	54.3 ± 13.6	*U* = 2,070.5, *r* = 0.02, *p* = 0.787
QADS – animal reminder	25.6 ± 10.1	28.8 ± 9.6	*U* = 1,634.0, *r* = −0.16, *p* = 0.069
QADS – contamination-intepersonal	42.0 ± 12.8	39.7 ± 10.3	*U* = 2,278.5, *r* = 0.11, *p* = 0.205
BPAQ – physical agression	19.4 ± 7.1	20.9 ± 7.3	*U* = 1,622.0, r = −0.09, p = 0.295
BPAQ – verbal agression	13.8 ± 5.3	12.4 ± 4.7	*U* = 2,082.0, r = 0.12, p = 0.185
BPAQ – anger	18.4 ± 6.3	19.5 ± 6.1	*U* = 1,648.5, r = −0.09, p = 0.363
BPAQ – hostility	19.0 ± 8.1	17.8 ± 7.7	*U* = 1,985.0, r = 0.07, p = 0.410
IVE – adventuresomeness	8.9 ± 3.4	8.9 ± 3.2	*U* = 1,931.0, r = −0.01, p = 0.886
IVE – empathy	12.3 ± 3.3	11.4 ± 3.5	*U* = 2,232.5, r = 0.12, p = 0.179
IVE – impulsivity	10.7 ± 4.2	8.5 ± 3.8	***U*** **= 2612.5**, ***r*** **= 0.29, p < 0.001**

*Data expressed as mean ± SD or n (%)*.

*Significant differences (p < 0.05) were marked with bold characters*.

*BPAQ, the Buss-Perry Aggression Questionnaire; CDI2, the Children's Depression Inventory 2; EI, emotional intelligence; IVE, the Eysenck's Impulsivity Inventory; PEIQ, the Popular Emotional Intelligence Questionnaire; QADS, the Questionnaire for the Assessment of Disgust Sensitivity; Self-harm (+), adolescents with a positive lifetime history of self-harm; Self-harm (–), adolescents with a negative lifetime history of self-harm; SES, the Rosenberg; Self-Esteem Scale; STAI, the State-Trait Anxiety Inventory*.

Table 2 shows bivariate correlations between EI and other measures tested in this study. There were significant negative associations between the level of EI (PEIQ – total score and scores of acceptance and control) and the scores of depressive symptoms and anxiety. Lower level of the PEIQ control subscale was related to significantly higher levels of core disgust. In turn, higher levels of the PEIQ acceptance subscale were associated with significantly higher levels of physical and verbal aggression, anger, hostility, venturesomeness as well as empathy. There were also significant negative correlations between the levels of impulsivity and the total PEIQ score as well as scores of two PEIQ subscales (control and understanding). Finally, higher levels of empathy (IVE) were significantly associated with the PEIQ total score and the scores of three PEIQ subscales (acceptance, control and empathy).

**Table 2:** Correlations between the level of emotional intelligence and other measures recorded in this study.

	**PEIQ – total score**	**PEIQ - acceptance**	**PEIQ – control**	**PEIQ – empathy**	**PEIQ - understanding**
CDI2 - depression	*r* = −0.350^b^	*r* = −0.519^c^	*r* = −0.369^b^	*r* = 0.156	*r* = −0.130
STAI - trait anxiety	*r* = −0.248^b^	*r* = −0.350^c^	*r* = −0.297^b^	*r* = 0.086	*r* = −0.077
STAI - state anxiety	*r* = −0.423^c^	*r* = −0.477^c^	*r* = −0.400^c^	*r* = 0.103	*r* = −0.205
SES - self-esteem	*r* = 0.345^c^	*r* = 0.382^c^	*r* = 0.300^b^	*r* = −0.068	*r* = 0.128
QADS - disgust (total score)	*r* = 0.071	*r* = 0.124	*r* = −0.142	*r* = 0.038	*r* = −0.076
QADS – core disgust	*r* = −0.048	*r* = −0.002	*r* = −0.268^b^	*r* = 0.103	*r* = −0.160
QADS – animal reminder	*r* = 0.018	*r* = 0.021	*r* = −0.167	*r* = 0.014	*r* = −0.119
QADS – contamination/interpersonal	*r* = −0.023	*r* = 0.056	*r* = −0.126	*r* = 0.100	*r* = −0.172
BPAQ - physical agression	*r* = 0.162	*r* = 0.282^b^	*r* = −0.077	*r* = 0.095	*r* = −0.143
BPAQ - verbal agression	*r* = 0.122	*r* = 0.191^a^	*r* = −0.082	*r* = 0.171	*r* = −0.052
BPAQ - anger	*r* = 0.102	*r* = 0.230^a^	*r* = 0.009	*r* = 0.081	*r* = 0.013
BPAQ - hostility	*r* = 0.077	*r* = 0.219^a^	*r* = −0.062	*r* = 0.121	*r* = −0.075
IVE - venturesomeness	*r* = 0.177^a^	*r* = 0.204^a^	*r* = −0.034	*r* = 0.171	*r* = 0.051
IVE - empathy	*r* = 0.266^b^	*r* = 0.192^a^	*r* = −0.182^a^	*r* = 0.518^c^	*r* = −0.168
IVE - impulsivity	*r* = −0.184^a^	*r* = −0.073	*r* = −0.437^c^	*r* = 0.111	*r* = −0.328^c^

*Spearman rank correlation coefficients were shown*.

a *p < 0.05*.

b *p < 0.01*.

c *p < 0.001*.

*BPAQ, the Buss-Perry Aggression Questionnaire; CDI2, the Children's Depression Inventory 2; EI, emotional intelligence; IVE, the Eysenck's Impulsivity Inventory; PEIQ, the Popular Emotional Intelligence Questionnaire; QADS, the Questionnaire for the Assessment of Disgust Sensitivity; SES, the Rosenberg Self-Esteem Scale; STAI, the State-Trait Anxiety Inventory*.

Results of mediation analysis were presented in Table 3. There were significant direct effects of EI on the level of depression (PEIQ – total score, PEIQ – acceptance score and PEIQ - control score), state and trait anxiety (PEIQ – total score, PEIQ – acceptance score and PEIQ - control score), impulsivity (PEIQ – total score, PEIQ – understanding score and PEIQ - control score) as well self-esteem (PEIQ – total score, PEIQ – acceptance score and PEIQ - control score). Similarly, direct effects of self-esteem, state and trait anxiety on a history of self-injuries were also significant in these models. No significant effects of depressive symptoms as mediators were found. Self-esteem, state and trait anxiety mediated the association between EI and a history of self-injuries in the models with the PEIW total scores as well as the scores of two subscales, including control and acceptance (significant indirect effects). Direct effects of EI on a history of self-injuries were non-significant in these models. Therefore, these results indicate that self-esteem, trait and state were complete mediators.

**Table 3:** Results of mediation analysis.

**Mediator**	**Effect**	**Predictor**			
		**PEIQ – total score**	**PEIQ - acceptance**	**PEIQ - control**	**PEIQ – understanding**
CDI2 - depression	Effect of EI on mediator (a)	B = −0.104^a^, SE = 0.047, 95% CI = −0.189 to −0.007	B = −0.523^c^, SE = 0.137, 95% CI = −0.750 to −0.253	B = −0.582^a^, SE = 0.202, 95% CI = −0.928 to −0.122	–
	Effect of mediator on self-injuries (b)	B = 0.001, SE = 0.002, 95% CI = −0.004 to 0.003	B = 0.004, SE = 0.007, 95% CI = −0.011 to 0.018	B = 0.006, SE = 0.006, 95% CI = −0.005 to 0.017	–
	Direct effect of EI on self-injuries (c)	B = −0.001, SE = 0.009, 95% CI = −0.018 to 0.016	B = 0.019, SE = 0.037, 95% CI = −0.047 to 0.085	B = −0.018, SE = 0.047, 95% CI = −0.110 to 0.075	–
	Indirect effect (ab)	B = −0.002, SE = 0.001, 95% CI = −0.005 to 0.001	B = −0.006, SE = 0.004, 95% CI = −0.014 to 0.002	B = −0.016, SE = 0.006, 95% CI = −0.029 to 0.004	–
STAI - state anxiety	Effect of EI on mediator (a)	B = −0.145^c^, SE = 0.032, 95% CI = −0.208 to −0.082	B = −0.480^c^, SE = 0.095, 95%CI = −0.659 to −0.288	B = −0.579^c^, SE = 0.170, 95% CI = −0.919 to −0.259	–
	Effect of mediator on self-injuries (b)	B = 0.015^b^, SE = 0.005, 95% CI = 0.006 to 0.025	B = 0.016^b^, SE = 0.005, 95% CI = 0.007 to 0.026	B = 0.014^b^, SE = 0.005, 95% CI = 0.005 to 0.024	–
	Direct effect of EI on self-injuries (c)	B = 0.001, SE = 0.001, 95% CI = −0.003 to 0.003	B = 0.001, SE = 0.005, 95% CI = −0.008 to 0.010	B = −0.049, SE = 0.034, 95% CI = −0.111 to 0.023	–
	Indirect effect (ab)	**B = −0.013, SE = 0.007,** **95% CI = −0.032 to −0.004**	**B = −0.045, SE = 0.023,** **95% CI = −0.104 to −0.015**	**B = −0.049, SE = 0.027,** **95% CI = −0.118 to −0.012**	–
STAI - trait anxiety	Effect of EI on mediator (a)	B = −0.093^b^, SE = 0.030, 95% CI = −0.149 to −0.031	B = −0.332^b^, SE = 0.003, 95% CI = −0.531 to −0.118	B = −0.444^b^, SE = 0.009, 95% CI = −0.780 to −0.130	–
	Effect of mediator on self-injuries (b)	B = 0.010^a^, SE = 0.004, 95% CI = 0.002 to 0.019	B = 0.010^a^, SE = 0.004, 95% CI = 0.002 to 0.019	B = 0.009^a^, SE = 0.004, 95% CI = 0.002 to 0.018	–
	Direct effect of EI on self-injuries (c)	B = −0.002, SE = 0.001, 95% CI = −0.004 to 0.001	B = −0.003, SE = 0.004, 95% CI = −0.011 to 0.006	B = −0.012, SE = 0.006, 95% CI = −0.024 to 0.001	–
	Indirect effect (ab)	**B = −0.006, SE = 0.004,** **95% CI = −0.016 to −0.001**	**B = −0.021, SE = 0.014,** **95%CI = −0.057 to −0.003**	**B = −0.026, SE = 0.019,** **95% CI = −0.076 to −0.002**	–
IVE - impulsivity	Effect of EI on mediator (a)	B = −0.025^a^, SE = 0.011, 95% CI = −0.046 to −0.004	–	B = −0.218^b^, SE = 0.001, 95% CI = −0.328 to −0.111	B = −0.219^c^, SE = 0.058, 95% CI = −0.328 to −0.101
	Effect of mediator on self-injuries (b)	B = 0.019, SE = 0.011, 95% CI = −0.002 to 0.040	–	B = 0.012, SE = 0.012, 95% CI = −0.011 to 0.035	B = 0.016, SE = 0.011, 95% CI = −0.006 to 0.039
	Direct effect of EI on self-injuries (c)	B = −0.002, SE = 0.001, 95% CI = −0.004 to 0.001	–	B = −0.014, SE = 0.007, 95% CI = −0.029 to 0.001	B = −0.010, SE = 0.007, 95% CI = −0.023 to 0.003
	Indirect effect (ab)	B = −0.002, SE = 0.002, 95% CI = −0.007 to 0.001	–	B = −0.014, SE = 0.014, 95% CI = −0.044 – 0.016	B = −0.017, SE = 0.014, 95% CI = −0.050 to 0.007
SES - self-esteem	Effect of EI on mediator (a)	B = 0.058^a^, SE = 0.022, 95% CI = 0.013 to 0.101	B = 0.206^b^, SE = 0.064, 95% CI = 0.078 to 0.329	B = 0.231^a^, SE = 0.099, 95% CI = 0.033 to 0.423	–
	Effect of mediator on self-injuries (b)	B = −0.018^b^, SE = 0.007 95% CI = −0.031 to −0.004	B = −0.019^b^, SE = 0.006, 95% CI = −0.032 to −0.005	B = −0.017^a^, SE = 0.007, 95% CI = −0.030 to −0.003	–
	Direct effect of EI on self-injuries (c)	B = −0.001, SE = 0.001, 95% CI = −0.004 to 0.001	B = −0.002, SE = 0.005, 95% CI = −0.011 to 0.008	B = −0.011, SE = 0.007, 95% CI = −0.025 to 0.001	–
	Indirect effect (ab)	**B = −0.005, SE = 0.003,** **95% CI = −0.013 to −0.001**	**B = −0.017, SE = 0.011,** **95% CI = −0.046 to −0.003**	**B = −0.019, SE = 0.013,** **95% CI = −0.049 to −0.003**	–

a *p < 0.05*.

b *p < 0.01*.

c *p < 0.001*.

*CDI2, the Children's Depression Inventory 2; EI, emotional intelligence; IVE, the Eysenck's Impulsivity Inventory; SES, the Rosenberg Self-Esteem Scale; STAI, the State-Trait Anxiety Inventory. Significant indirect effects were marked with bold characters*.

## Discussion

Results of this study imply that individuals with conduct disorder and positive lifetime history of self-injuries present with significantly lower levels of EI and self-esteem together with higher levels of depressive and anxiety symptoms as well as impulsivity. Previous studies have also shown that on the one hand depression is associated with a higher risk of self-harm (36) as well as with lower level of EI on the other hand. A negative correlation between the level of EI or its components and depressive symptoms score has been replicated in early, middle and late adolescence (37–39). Regarding anxiety, similar results have been shown. In a cross-sectional study conducted in over 12,000 adolescents from 11 European countries, it was demonstrated that not only depression but also anxiety symptoms are significantly associated with self-harm risk (40). Furthermore, self-reported EI was negatively correlated with anxiety severity, social anxiety and the level of stress in adolescent samples (41, 42). Moreover, consistent findings have been reported with respect to impulsivity. Chamberlain et al. (4) found that self-harm dimensions are associated with impulse control disorders. A higher level of impulsiveness has previously been found in subjects with a history of self-injuring (12, 23). Finally, people with higher levels of EI are characterized by less frequent engagement in self-harm acts (20, 40), less frequent suicide attempts (43) and better overall social functioning (40). These observations appear to be consistent and independent of age (40), cultural context (12), nationality (44) or self-harm method (2). Therefore, high EI level might be perceived as a protective factor for self-harm.

One of the most important variables associated with self-harm risk is self-esteem. Greydanus and Shek (45) found that adolescents with low levels of self-esteem are at higher risk of engaging in self-injuries. A large number of previous reviews have consistently shown links between self-harm behaviors and low levels of self-respect among adolescents (7, 9). Hodgson (46) demonstrated that those who reported self-harm have also more problems with self-criticism and self-denigration. Moreover, they tend to present lower levels of self-esteem in contrast to adolescents who never engaged in self-harm acts. Increased self-dislike also advocates for the concept of self-harm as a way of punishing oneself and growing self-hatred of one's own body (25).

We also found that higher levels of EI are related to higher levels of self-esteem, venturesomeness and empathy, and at the same time with lower levels of depressive symptoms, anxiety and impulsivity in adolescents with conduct disorder. High levels of EI have been reported in correlation with a lower severity of symptoms related to mood and anxiety disorders (17, 27). High level of EI has been related to a subjective perception of well-being and satisfaction with life as well as higher levels of self-esteem (47). In some studies, lower self-esteem has been associated with a higher frequency of self-injuries (46). Importantly, self-esteem has also been found to mediate the association between childhood maltreatment and self-injuries in adolescents (47).

Similar results have been reported with respect to impulsiveness. It has been found that higher levels of impulsiveness are linked with a risk of self-harm. Moreover, there is evidence that self-injuries are driven by a wish to lessen emotional distress, and increased negative affect may precede episodes of self-harm (48). Higher level of impulsivity has been identified in individuals with self-harm history, because they worry less about the long-term consequences (e.g., discomfort, scarring, stigmatization). They can also be encouraged to self-injurious behavior by the promise of the immediate benefits (e.g., relief) (49, 50). Notably, we did not find any significant association between self-injuries and disgust sensitivity. Higher levels of core disgust were weakly associated with lower levels of control over one's emotions. It was previously demonstrated in college students that another type of disgust referred to as self-disgust plays a role as a mediator between depressive symptoms and NSSI (51). However, this category of disgust was not included in our study.

Our path analysis demonstrated that trait and state anxiety as well as impulsivity completely mediate the association between EI and a lifetime history of self-injuries in this group of adolescents (non-significant direct effects on a history of self-injuries with significant indirect effects). Previous studies have also shown that EI is not directly related to a risk of self-injuries or suicide. For instance, a recent study by Quintana-Orts et al. (19) demonstrated that depressive symptoms mediate the association between suicide risk and EI among victims of bullying. This effect was moderated by sex, and appeared to be stronger in girls compared to boys. It is important to note that we did not find that depressive symptoms mediate the association between EI and a risk of self-injuries. However, to the best of our knowledge, our study is the first which was performed in adolescents with conduct disorder and we focused on a risk of self-injuries. Similarly, another study demonstrated that the level of psychological distress mediates the relationship between EI and suicide risk in adults (52). In turn, (53, 54) revealed that recognition and expression of emotions mediate the association between mindfulness and distress. The same study provided evidence that emotional recognition and expression as well as emotional management and control mediate the association between mindfulness and depression in adolescents.

There are some limitations of this research that need to be addressed. Our sample was rather small and a type II error cannot be ignored. Similarly, type I error should be taken into consideration due to a large number of estimated effects and a lack of correction for multiple testing. Therefore, our findings should be perceived as exploratory and requiring independent verification. Moreover, a cross-sectional study design does not support causal associations. Indeed, it has been demonstrated that the cross-sectional approaches can generate biased estimates of associations that are hypothesized to have a temporal ordering (55). Moreover, our findings cannot be generalized to other clinical populations with high prevalence of self-injuries. Although previous studies indicate that various psychological processes and low emotional abilities precede depressive symptoms, anxiety and self-harm behaviors, longitudinal studies are needed to investigate validity of the model tested in our study. Another limitation is that two subscales of the IVE (venturesomeness and empathy) had questionable internal consistency. Finally, investigating our hypotheses in a specific group of adolescents with conduct disorder limits generalization of findings to other populations.

In conclusion, main findings of our studies indicate that EI is not directly associated with a risk of self-injuries in adolescents with conduct disorder. Anxiety and self-esteem might serve as complete mediators of this association. However, longitudinal studies are required to confirm direction of causality. Results of our study hold a great promise for developing specific interventions that aim to target or prevent self-injurious behaviors. In light of our findings, one of potential approaches would be to target emotional competences of vulnerable individuals (43–50). Moreover, focusing on the development of self-esteem and reducing the level of anxiety seems to have an important role.

## Data Availability Statement

The raw data supporting the conclusions of this article will be made available by the authors, without undue reservation.

## Ethics Statement

The studies involving human participants were reviewed and approved by Ethics Committee of Wroclaw Medical University, Poland. Written informed consent to participate in this study was provided by the participants' legal guardian/next of kin.

## Author Contributions

JH-M collected data and wrote the first draft of the manuscript. MS-B participated in data analysis and manuscript writing. JR participated in manuscript writing. AA participated in data collection and manuscript writing. BM performed data analysis and participated in manuscript writing. All authors contributed to the article and approved the submitted version.

## Conflict of Interest

The authors declare that the research was conducted in the absence of any commercial or financial relationships that could be construed as a potential conflict of interest.
